# Drug-resistant Salmonella enterica serotype paratyphi A in India.

**DOI:** 10.3201/eid0604.000420

**Published:** 2000

**Authors:** D S Chandel, R Chaudhry, B Dhawan, A Pandey, A B Dey

**Affiliations:** All India Institute of Medical Sciences, Ansari Nagar, New Delhi, India.

## Abstract

The incidence of enteric fever caused by Salmonella enterica serotype Paratyphi A has been increasing in India since 1996. In 1998, the incidence of enteric fever caused by drug- resistant S. Paratyphi A abruptly increased in the New Delhi region. In the first 6 months of 1999, 32% of isolates were resistant to both chloramphenicol and cotrimoxazole and another 13% were resistant to more than two antibiotics.


### Drug-Resistant *Salmonella enterica* Serotype Paratyphi A in India

**Dinesh S. Chandel, Rama Chaudhry, Benu Dhawan, Anita Pandey, Aparajit B. Dey**
All India Institute of Medical Sciences,
Ansari Nagar, New Delhi, India

The incidence of enteric fever caused by *Salmonella enterica* serotype Paratyphi A has been increasing in India since 1996. In 1998, the incidence of enteric fever caused by drug-resistant *S.* Paratyphi A abruptly increased in the New Delhi region. In the first 6 months of 1999, 32% of isolates were resistant to both chloramphenicol and cotrimoxazole and another 13% were resistant to more than two antibiotics.

Enteric fever (typhoid) is classically caused by *Salmonella enterica* serotype Typhi, but a similar syndrome may be observed with *S.* Paratyphi A and other serotypes. Outbreaks of enteric fever associated with *S.* Paratyphi A have rarely been reported in India (1–3). Although multidrug-resistant outbreaks of *S.* Typhi with an increase in numbers of strains with decreased susceptibility to ciprofloxacin have occurred, cases of drug-resistant *S.* Paratyphi A have been relatively uncommon (2). We report a sudden increase in enteric cases caused by drug-resistant *S.* Paratyphi A unresponsive to ciprofloxacin therapy.

### The Study

We screened all recent isolates of *S.* Paratyphi A from hospitals in Delhi and adjoining areas for susceptibility (MICs) to various drugs. A total of 105 sporadic isolates of *S.* Paratyphi A from All India Institute of Medical Sciences (67 isolates), Safdarjang Hospital (31 isolates), New Delhi and Rohtak Medical College, Haryana (7 isolates) (an Indian state near New Delhi) were collected from April 1996 to July 1999 and tested for susceptibility to chloramphenicol, cotrimoxazole, amoxicillin, and ciprofloxacin by comparative disc diffusion (4). MICs to ciprofloxacin were estimated by E-test (AB-Biodisc, Sweden) according to guidelines from the National Committee for Clinical Laboratory Standards (NCCLS).

Address for correspondence: Rama Chaudhry, Department of Microbiology, All India Institute of Medical Sciences, New Delhi-110029; fax: 91-11-6862663; e-mail: rc123@hotmail.com.

In the study period, *S.* Paratyphi A isolations in enteric fever cases were 10, 16, 57, and 22, in 1996 (April), 1997, 1998, and 1999 (through July), respectively. During 1996-97, isolates were uniformly susceptible to all antibiotics, including ciprofloxacin and ceftriaxone, commonly used in the treatment of enteric fever. However, in 1998, the incidence of enteric fever caused by drug-resistant *S.* Paratyphi A abruptly increased (up to 24% of isolates), and the number of drug-resistant isolates susceptible to ciprofloxacin markedly decreased. MICs of 0.25 to 1.5 mg/L were recorded (Table). In the first 6 months of 1999, 7 (32%) of 22 isolates were resistant to both chloramphenicol and cotrimoxazole and another 3 (13%) were resistant to more than two drugs. Compared with isolates from 1996 to 1998, most drug-resistant isolates in 1999 showed higher MICs to ciprofloxacin.

### Conclusions

*S.* Paratyphi A, which causes 1%-15% of enteric fever cases in India, has been increasing since 1996 (3). Our study found that 32% of isolates from the New Delhi region had decreased susceptibility to ciprofloxacin (MIC >2.0 mg/L), the drug of choice for enteric fever in India. One sequela of this increased resistance was delay in the resolution of symptoms. Although strains may appear sensitive at this level, when subjected to ciprofloxacin-susceptibility testing by disc diffusion, treatment failure may still occur.

The mechanisms proposed for quinolone resistance involve alteration in the permeability of the drug (outer membrane protein gene

Table. Resistance pattern and ciprofloxacin MIC of *Salmonella* Paratyphi A isolates, New Delhi, India, 1996–1999

| | | Drug-resistance pattern | | | | | | | Ciprofloxacin MIC | |
|---|---|---|---|---|---|---|---|---|---|---|
| Year | Strains (no.) | Cl | Cz | Ax | Cp | Cl+ Cz | Cl+ Cz+ Ax | Total (%) | Range (mg/L) | Total (%) |
| 1996 | 10 | - | - | - | - | 1 | - | 1 (10) | <0.0025 | - |
| 1997 | 16 | 2 | - | - | - | 1 | - | 3 (18) | <0.045 | - |
| 1998 | 57 | - | 11 | - | - | - | 3 | 14 (24) | 0.25-1.5 | 12 (21) |
| 1999[a] | 22 | - | - | - | - | 7 | 3 | 10 (45) | 2.0 | 7 (32) |

[a]until July 1999.
- = sensitive range; Cl=chloramphenicol; Cz=cotrimoxazole; Ax=amoxicillin; Cp=ciprofloxacin.

mutation) or alteration of the target enzyme DNA gyrase (5) within the treated bacterium as its adaptive reflex. Since resistance to quinolones is independent of resistance to other drugs that are mainly plasmid mediated, it may occur in otherwise sensitive strains. Similar R-plasmids of the IncHi Group have been documented: four strains of drug-resistant *S.* Paratyphi A were shown to harbor such plasmids encoding transferrable resistance to many drugs (ampicillin, chloramphenicol, sulfamethoxazole, and tetracycline) other than ciprofloxacin (6). The incidence of plasmids conferring multidrug resistance is increasing in *Salmonella* serotypes, including Enterobacteriaceae, where transfer of these R-plasmids to *S.* Paratyphi A strains may have occurred. Continuous surveillance for the susceptibility patterns of current isolates is needed.

However, development of resistance to ciprofloxacin has been suggested as partly related to exposures of these organisms to concentrations near their MICs. With increases in MICs, clinicians may be tempted to administer higher doses of ciprofloxacin to achieve serum levels required for effective therapy; however, higher doses could have unwanted clinical and public health consequences. Rather, this increased resistance may warrant a restructuring of the chemotherapeutic regimen for enteric diseases, as well as restricting use of ciprofloxacin to atypical cases in which lack of clinical response to other therapeutic drugs is noted.

Chloramphenicol and amoxicillin may need to be reconsidered as the drugs of choice in cases of enteric fever because of the increased susceptibilities of such cases to these drugs (>90% for reemerging isolates of *S.* Typhi [3]). However, these recommendations might not be appropriate in view of the substantial increase in drug-resistant *S.* Paratyphi A infections, which often obfuscate the clinical diagnosis and management of enteric fever. The increase in incidence of enteric fever caused by *S.* Paratyphi A could possibly be related to widespread use of vaccines and quinolones against *S.* Typhi in the past decade. Regardless, the frequency and geographic diversity of these cases increase the potential for large outbreaks of drug-resistant *S.* Paratyphi A in India.

### Acknowledgments

We acknowledge P. Seth for providing facilities and support; A. Kapil, H. Kapoor, and D.R. Arora for their help; and P. Arya for technical assistance.

This work was supported by the Department of Biotechnology (DO#BT/R&D/9/11/94), Ministry of Science & Technology, New Delhi, India.

Dr. Chandel is pursuing his Ph.D. in medical microbiology at the All India Institute of Medical Sciences, New Delhi, under the direction of Dr. R. Chaudhry. His research interests include molecular diagnostics and typing of bacterial pathogens, especially characterizing *Salmonella* in enteric diseases, and antimicrobial resistance mechanisms.

### References

1. Thong KL, Nair S, Chaudhry R, Seth P, Kapil A, Chandel DS, et al. Molecular analysis of *Salmonella* Paratyphi A from an outbreak in New Delhi, India, by ribotyping and pulsed-field gel electrophoresis. Emerg Infect Dis 1998;4:507-8.
2. Mahanta J. Drug sensitivity of Salmonella paratyphi A isolated from a suspected outbreak of enteric fever in Duliajan. J Indian Med Assoc 1994;92:49-50.
3. Sood S, Kapil A, Dash N, Das BK, Goel V, Seth P, et al. Paratyphoid fever in India: An emerging problem. Emerg Infect Dis 1999;5:483-4.
4. Stokes EJ, Ridgway GL. Clinical bacteriology: antibacterial drugs. 5th ed. London: Edward Arnold; 1980. p.205-19.
5. Cullman W, Steieglitz M, Bams B, Opferkuch W. Comparative evaluation of newly developed quinolone compounds, with a note on the frequency of resistant mutants. Chemotherapy 1985;31:19-28.
6. Rangnekar VM, Banker DD, Jhala HI. Antimicrobial resistance and R-plasmids of Salmonella paratyphi A isolated in Bombay. Indian J Med Res 1983;77;5-9.
